# 2,5-Dichloro­thio­phene 1,1-dioxide

**DOI:** 10.1107/S1600536809050776

**Published:** 2009-11-28

**Authors:** Jonathan B. Briggs, Wenling Jia, Mikaël D. Jazdzyk, Glen P. Miller

**Affiliations:** a Department of Chemistry and Materials Science Program, University of New Hampshire, Durham, NH 03824-3598, USA

## Abstract

The complete mol­ecule of the title compound, C_4_H_2_Cl_2_O_2_S, is generated by crystallographic twofold symmetry, with the S atom lying on the rotation axis. In the crystal, the molecules are linked by C—H⋯O hydrogen bonds..

## Related literature

For a related thio­phene-1,1-dioxide structure, see: Douglas *et al.* (1993 ▶). For the synthetic utility and related applications of thio­phene-1,1-dioxides, see: Moiseev *et al.* (2006 ▶); Nakayama & Sugihara (1999 ▶); Shul’ts *et al.* (2003 ▶); Lou *et al.* (2002 ▶).
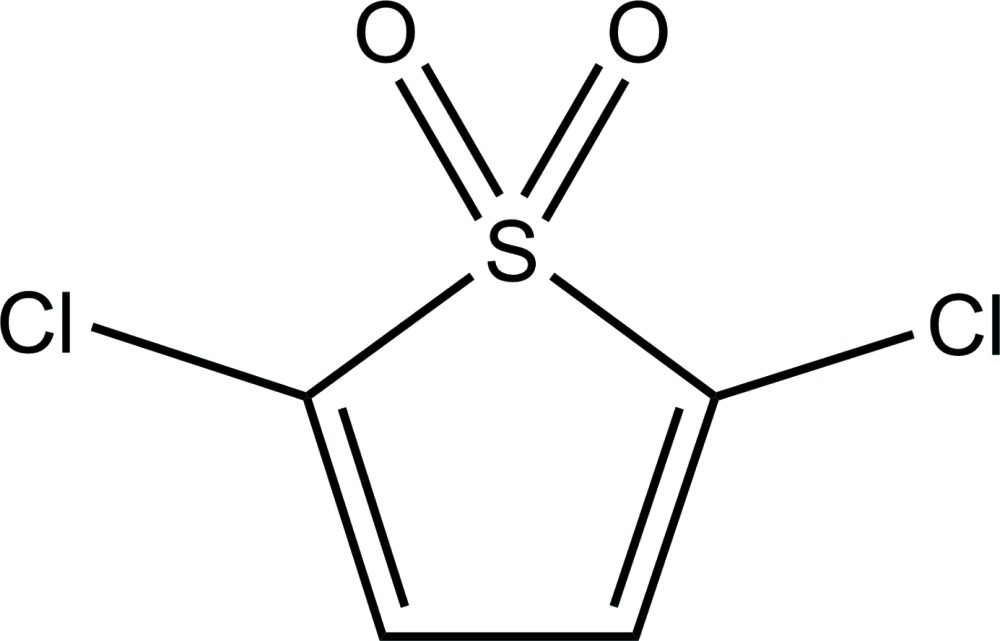


## Experimental

### 

#### Crystal data

C_4_H_2_Cl_2_O_2_S
*M* *_r_* = 185.02
Monoclinic, 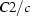

*a* = 7.588 (2) Å
*b* = 10.584 (3) Å
*c* = 8.745 (3) Å
β = 90.275 (9)°
*V* = 702.4 (3) Å^3^
*Z* = 4
Mo *K*α radiation
μ = 1.14 mm^−1^
*T* = 296 K
0.50 × 0.40 × 0.30 mm

#### Data collection

Bruker SMART X2S diffractometer
Absorption correction: multi-scan (*SADABS*; Bruker, 2007 ▶) *T*_min_ = 0.590, *T*_max_ = 0.726
3352 measured reflections
622 independent reflections
549 reflections with *I* > 2σ(*I*)
*R*_int_ = 0.028

#### Refinement

*R*[*F* ^2^ > 2σ(*F* ^2^)] = 0.035
*wR*(*F* ^2^) = 0.094
*S* = 1.12
622 reflections
42 parameters
H-atom parameters constrained
Δρ_max_ = 0.21 e Å^−3^
Δρ_min_ = −0.31 e Å^−3^

### 

Data collection: *GIS* (Bruker, 2007 ▶); cell refinement: *SAINT* (Bruker, 2007 ▶); data reduction: *SAINT* (Bruker, 2007 ▶); program(s) used to solve structure: *SHELXS97* (Sheldrick, 2008 ▶); program(s) used to refine structure: *SHELXL97* (Sheldrick, 2008 ▶); molecular graphics: *SHELXTL* (Sheldrick, 2008 ▶); software used to prepare material for publication: *SHELXTL*.

## Supplementary Material

Crystal structure: contains datablocks I, global. DOI: 10.1107/S1600536809050776/fl2274sup1.cif

Structure factors: contains datablocks I. DOI: 10.1107/S1600536809050776/fl2274Isup2.hkl

Additional supplementary materials:  crystallographic information; 3D view; checkCIF report

## Figures and Tables

**Table 1:** Hydrogen-bond geometry (Å, °)

*D*—H⋯*A*	*D*—H	H⋯*A*	*D*⋯*A*	*D*—H⋯*A*
C2—H2⋯O1^i^	0.93	2.52	3.367 (4)	152

Symmetry code: (i) 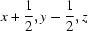.

**Fig. 1.**
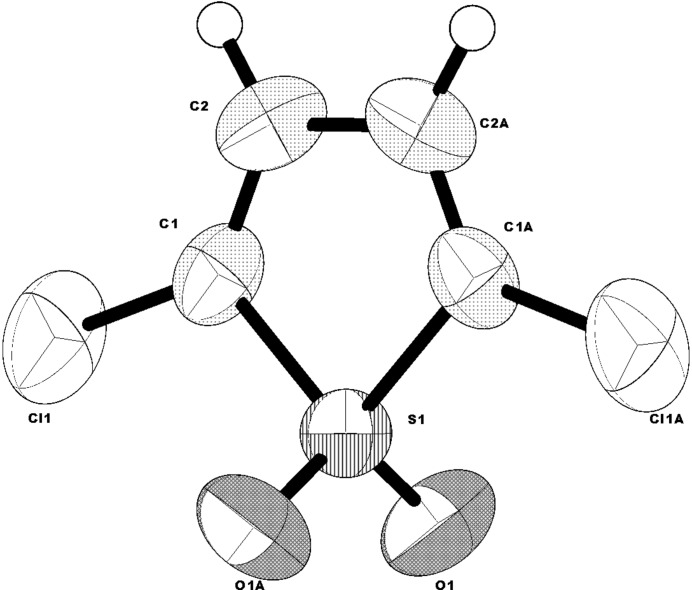
The molecular structure showing the crystallographic labelling scheme and displacement ellipsoids drawn at the 50% probability level. H atoms are shown as spheres of arbitrary radii.

**Fig. 2.**
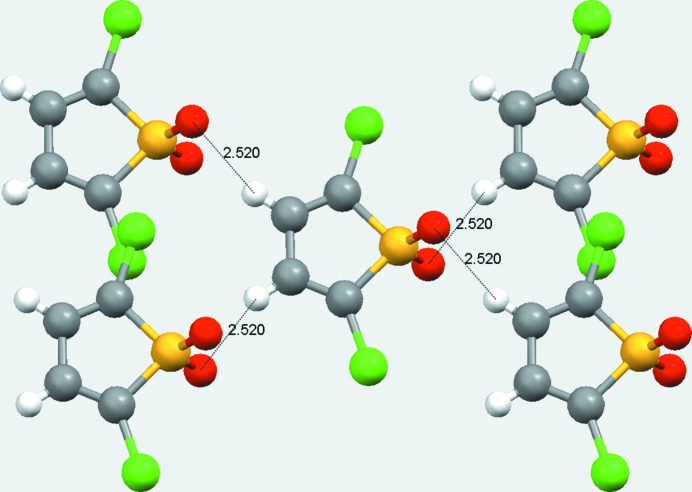
Perspective view of the title compound showing sets of identical H-bonds.

**Fig. 3.**
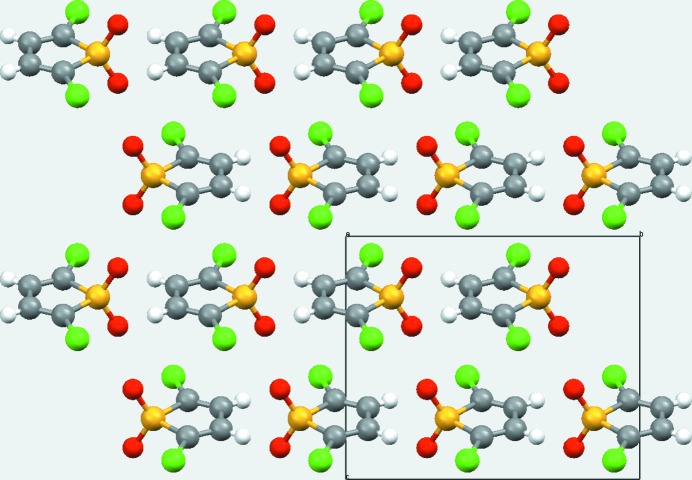
Perspective view of the title compound looking down the a axis of the unit cell.

**Fig. 4.**
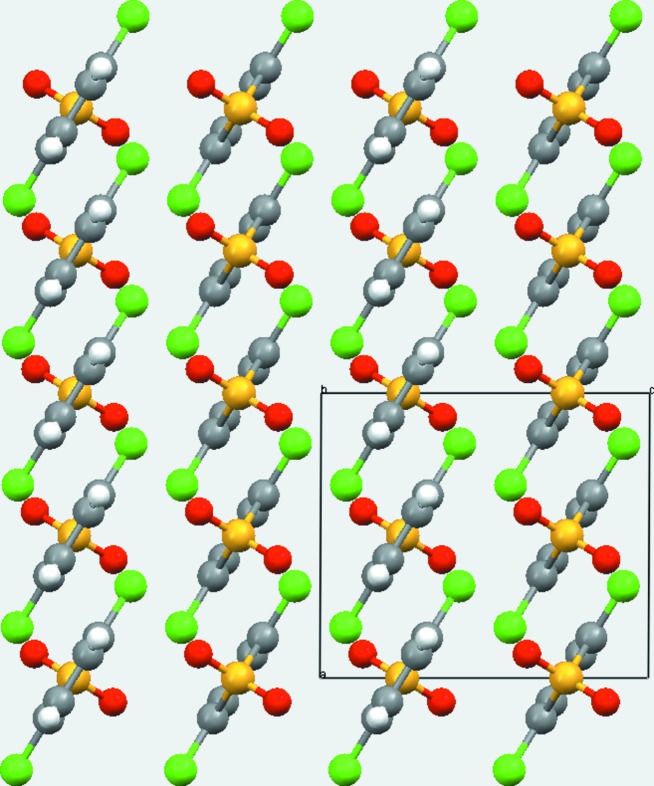
Perspective view of the title compound looking down the b axis of the unit cell.

**Fig. 5.**
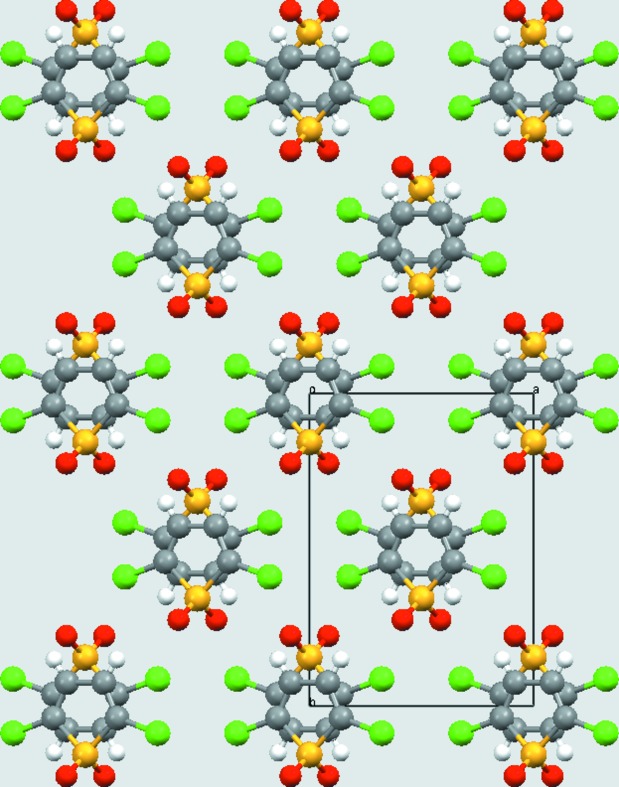
Perspective view of the title compound looking down the c axis of the unit cell.

**Fig. 6.**
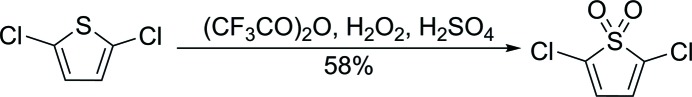
Synthesis of the title compound, 2,5-dichlorothiophene-1,1-dioxide.

## supplementary crystallographic information

### Comment

Thiophene 1,1-dioxides are important building blocks in modern organic synthesis and materials chemistry (Moiseev *et al.*, 2006). In particular, thiophene 1,1-dioxides have been utilized as Diels-Alder dienes in the construction of larger molecules (Nakayama & Sugihara, 1999) including biologically active compounds (Shul'ts *et al.*, 2003) and halogenated derivatives (Lou *et al.*, 2002). The crystal structure for tetrachlorothiophene-1,1-dioxide has also been solved (Douglas *et al.*, 1993).

Figure 1 shows the displacement ellipsoid diagram with appropriate atomic labels. Although there is only one unique H-bonding interaction in the crystal structure (Figure 2), each molecule is in fact linked to four others through symmetry related versions of this same H-bond (Table 1). Each sulfone oxygen (O1 and O1A) H-bonds to one hydrogen atom (*i.e.*, O1 – H, 2.520 (2) Å; O1A – H, 2.520 (2) Å). Likewise, each hydrogen atom H-bonds to one sulfone oxygen atom. The sulfone groups do not interdigitate with the methines of an adjacent molecule.

Each unit cell contains two complete molecules. Looking down the *a* axis of the unit cell (Figure 3), the molecules in the crystal structure are arranged head to tail (with the sulfone end being the head) in horizontal rows, with alternating rows of inverted directionality (*i.e.*, sulfone head groups pointing "right" in one row and pointing "left" in adjacent rows). Likewise, looking down the *b* axis of the unit cell (Figure 4), the molecules are arranged head to tail in vertical columns, with alternating columns of inverted directionality (*i.e.*, sulfone head groups pointing "forward" in one column and pointing "backward" in adjacent columns). The inverted directionality between adjacent rows in Figure 3 and adjacent columns in Figure 4 is further illustrated upon looking down the *c* axis (Figure 5) where one observes molecular stacks with alternating up-down orientations of the sulfone head groups. This arrangement of molecules is driven by the 4:1 H-bonding network, as previously noted. However, relatively weak π-π stacking interactions also influence the arrangement of molecules, albeit to a lesser extent. Carbon atoms of closest contact in the molecular stacks are approximately 4.1 Å apart (Table 2) indicating weak π-π stacking interactions (the interlayer spacing in graphite is 3.4 Å) that can only be minimally responsible for the observed ordering of molecules.

### Experimental

The title compound was prepared according to a related literature procedure (Lou *et al.*, 2002) as illustrated in Figure 6. Thus, 2,5-dichlorothiophene was oxidized in 58% yield using a mixture of trifluoroacetic anhydride, hydrogen peroxide and sulfuric acid. The title compound was purified by silica gel column chromatography (30% dichloromethane - 70% hexane eluent). ^1^H NMR (400 MHz, CDCl_3_) δ 6.74 (s, 2H); ^13^C NMR (100 MHz, CDCl_3_) δ 123.45 (CH), 131.08 (CCl). An X-ray grade crystal was obtained by slow evaporation of a dichloromethane-hexane solution.

### Crystal data

**Table:**

C_4_H_2_Cl_2_O_2_S	*F*(000) = 368
*M**_r_* = 185.02	*D*_x_ = 1.750 Mg m^−^^3^
Monoclinic, *C*2/*c*	Mo *K*α radiation, λ = 0.71073 Å
Hall symbol: -C 2yc	Cell parameters from 1863 reflections
*a* = 7.588 (2) Å	θ = 3.3–24.8°
*b* = 10.584 (3) Å	µ = 1.14 mm^−^^1^
*c* = 8.745 (3) Å	*T* = 296 K
β = 90.275 (9)°	Block, colourless
*V* = 702.4 (3) Å^3^	0.50 × 0.40 × 0.30 mm
*Z* = 4	

### Data collection

**Table:**

Bruker SMART X2S diffractometer	622 independent reflections
Radiation source: micro-focus sealed tube	549 reflections with *I* > 2σ(*I*)
doubly curved silicon crystal	*R*_int_ = 0.028
ω scans	θ_max_ = 25.0°, θ_min_ = 3.3°
Absorption correction: multi-scan (*SADABS*; Bruker, 2007)	*h* = −9→8
*T*_min_ = 0.590, *T*_max_ = 0.726	*k* = −12→12
3352 measured reflections	*l* = −10→10

### Refinement

**Table:**

Refinement on *F*^2^	Primary atom site location: structure-invariant direct methods
Least-squares matrix: full	Secondary atom site location: difference Fourier map
*R*[*F*^2^ > 2σ(*F*^2^)] = 0.035	Hydrogen site location: inferred from neighbouring sites
*wR*(*F*^2^) = 0.094	H-atom parameters constrained
*S* = 1.12	*w* = 1/[σ^2^(*F*_o_^2^) + (0.0473*P*)^2^ + 0.6008*P*] where *P* = (*F*_o_^2^ + 2*F*_c_^2^)/3
622 reflections	(Δ/σ)_max_ = 0.013
42 parameters	Δρ_max_ = 0.21 e Å^−^^3^
0 restraints	Δρ_min_ = −0.31 e Å^−^^3^

### Special details

**Table:**

Geometry. All e.s.d.'s (except the e.s.d. in the dihedral angle between two l.s. planes) are estimated using the full covariance matrix. The cell e.s.d.'s are taken into account individually in the estimation of e.s.d.'s in distances, angles and torsion angles; correlations between e.s.d.'s in cell parameters are only used when they are defined by crystal symmetry. An approximate (isotropic) treatment of cell e.s.d.'s is used for estimating e.s.d.'s involving l.s. planes.
Refinement. Refinement of *F*^2^ against ALL reflections. The weighted *R*-factor *wR* and goodness of fit *S* are based on *F*^2^, conventional *R*-factors *R* are based on *F*, with *F* set to zero for negative *F*^2^. The threshold expression of *F*^2^ > σ(*F*^2^) is used only for calculating *R*-factors(gt) *etc*. and is not relevant to the choice of reflections for refinement. *R*-factors based on *F*^2^ are statistically about twice as large as those based on *F*, and *R*- factors based on ALL data will be even larger.

### Fractional atomic coordinates and isotropic or equivalent isotropic displacement parameters (Å^2^)

**Table:**

	*x*	*y*	*z*	*U*_iso_*/*U*_eq_	
Cl1	0.32276 (10)	0.08665 (9)	0.07774 (10)	0.0906 (4)	
S1	0.0000	0.15554 (8)	0.2500	0.0529 (3)	
O1	−0.0844 (3)	0.2242 (2)	0.1299 (2)	0.0779 (6)	
C1	0.1415 (3)	0.0379 (3)	0.1743 (3)	0.0582 (6)	
C2	0.0827 (4)	−0.0754 (3)	0.2059 (3)	0.0677 (8)	
H2	0.1398	−0.1490	0.1757	0.081*	

### Atomic displacement parameters (Å^2^)

**Table:**

	*U*^11^	*U*^22^	*U*^33^	*U*^12^	*U*^13^	*U*^23^
Cl1	0.0634 (5)	0.1157 (8)	0.0932 (6)	0.0154 (4)	0.0365 (4)	0.0078 (5)
S1	0.0513 (5)	0.0506 (5)	0.0570 (5)	0.000	0.0174 (4)	0.000
O1	0.0812 (13)	0.0728 (12)	0.0799 (13)	0.0258 (11)	0.0188 (10)	0.0181 (10)
C1	0.0518 (14)	0.0674 (15)	0.0553 (14)	0.0134 (12)	0.0115 (11)	0.0005 (12)
C2	0.0788 (19)	0.0587 (15)	0.0657 (17)	0.0167 (14)	0.0042 (14)	−0.0020 (13)

### Geometric parameters (Å, °)

**Table:**

Cl1—C1	1.698 (3)	S1—C1^i^	1.774 (2)
S1—O1^i^	1.427 (2)	C1—C2	1.310 (4)
S1—O1	1.427 (2)	C2—C2^i^	1.476 (6)
S1—C1	1.774 (2)	C2—H2	0.9300
			
O1^i^—S1—O1	118.8 (2)	C2—C1—Cl1	131.4 (2)
O1^i^—S1—C1	111.19 (13)	C2—C1—S1	110.9 (2)
O1—S1—C1	110.68 (12)	Cl1—C1—S1	117.74 (16)
O1^i^—S1—C1^i^	110.68 (12)	C1—C2—C2^i^	113.68 (16)
O1—S1—C1^i^	111.19 (13)	C1—C2—H2	123.2
C1—S1—C1^i^	90.87 (18)	C2^i^—C2—H2	123.2

Symmetry codes: (i) −*x*, *y*, −*z*+1/2.

### Hydrogen-bond geometry (Å, °)

**Table:**

*D*—H···*A*	*D*—H	H···*A*	*D*···*A*	*D*—H···*A*
C2—H2···O1^ii^	0.93	2.52	3.367 (4)	152

Symmetry codes: (ii) *x*+1/2, *y*−1/2, *z*.

### Table 2 π-π stacking interaction geometry (Å, °)

**Table:**

X···Y	π···π
C1···C2	4.137 (4)
